# Shared molecular mechanisms of common differentially expressed genes (C-DEGs) linking gallbladder stones to carcinogenesis: integrated bioinformatics and experimental validation

**DOI:** 10.1186/s12935-026-04193-7

**Published:** 2026-03-06

**Authors:** Jingzhe Yu, Yongchao Zhu, Tuoyu Lu, Yingzi Yu, Dong Jin, Genwang Wang, Xiangyang Wu, Zhenhui Lu

**Affiliations:** 1 Department of Pediatric Surgery, General Hospital of Ningxia Medical University, Yinchuan, 750004 China; 2 Institute of Medical Sciences, General Hospital of Ningxia Medical University, Yinchuan, 750004 China; 3 School of Pharmacy, Xi ’an Jiaotong University Health Science Center, Yinchuan, 750004 China; 4 hospital sensory department, Shenzhen Qianhai Shekou Free Trade Zone Hospital, Shenzhen, 518000 China; 5 Department of Hepatobiliary Surgery, General Hospital of Ningxia Medical University, Yinchuan, 750004 China; 6 General Hospital of Ningxia Medical University, 804 Shengli Street, Xingqing District, Yinchuan, 750004 Ningxia China; 7 Baoji City central Hospital liver, Baoji, 721000 China

**Keywords:** Gallbladder stone, Gallbladder carcinoma, Transcription factors (TFs), JAK-STAT signaling pathway

## Abstract

**Introduction:**

Gallbladder stones (GBS) and gallbladder cancer (GBC) are prevalent gallbladder diseases that, while presenting distinct clinical manifestations, may share common regulatory mechanisms at the molecular level. This study aimed to identify and validate common differentially expressed genes (C-DEGs) in GBS and GBC through comprehensive bioinformatics analysis and experimental validation to elucidate potential molecular pathological mechanisms linking these two conditions.

**Methods:**

We analyzed gene expression data from publicly available databases for GBS and GBC, identifying 94 common differentially expressed genes (C-DEGs) through differential expression analysis. Gene ontology (GO) functional analysis and Kyoto Encyclopedia of Genes and Genomes (KEGG) pathway analysis were performed to characterize the functions and pathways associated with these genes. Furthermore, we examined the differential expression of transcription factors (TFs) in both GBS and GBC groups and experimentally validated the functions of these genes.

**Results:**

Our analysis identified 94 C-DEGs, including 10 hub genes (HC-DEGs): SOCS3, GADD45B, SGK1, MYC, HBEGF, KLF10, EGF, IL6, NR4A3, and CDKN1A. GO functional analysis revealed that these genes primarily regulate smooth muscle cell proliferation, animal organ regeneration, peptidyl-tyrosine phosphorylation, epidermal growth factor-activated receptor activity, and the JAK-STAT signaling pathway. KEGG pathway analysis demonstrated their involvement in multiple cancers (bladder, endometrial, colorectal, thyroid, breast, and gastric), the FoxO signaling pathway, the JAK-STAT signaling pathway, the ErbB signaling pathway, and transcriptional dysregulation in cancer. Additionally, we identified 14 differentially expressed TFs in the GBS group and 11 in the GBC group. Four transcription factors—E2F1, ETS2, EZH2, and MYC—showed differential co-expression in both conditions and were jointly involved in regulating two hub genes (CDKN1A and MYC).

**Discussion:**

Through comprehensive bioinformatics analysis and experimental validation, this study revealed common molecular mechanisms between GBS and GBC. We identified and validated 10 hub genes that exhibited significant differential expression in both conditions and were primarily involved in multiple cancer-related signaling pathways. Furthermore, we identified four transcription factors (E2F1, ETS2, EZH2, and MYC) showing differential co-expression in both GBS and GBC, which jointly regulated two hub genes (CDKN1A and MYC). These findings illuminate shared molecular mechanisms between GBS and GBC, providing novel insights for further investigation of their molecular pathological mechanisms and establishing an important theoretical foundation for future preventive and therapeutic strategies.

## Introduction

Gallbladder stones (GBS) are a common gallbladder disease, primarily manifesting as cholesterol stones, cholesterol-based mixed stones, or melanin stones [1]. GBS predominantly affects adults, with increasing incidence after age 40 and higher prevalence among women [2]. Notably, geographical regions with high GBS incidence also demonstrate elevated rates of gallbladder carcinoma (GBC) [3]. Epidemiological studies have established a significant correlation between these conditions, with GBS patients exhibiting a 13.7-fold higher risk of developing GBC compared to individuals without GBS. Moreover, patients with single gallstones exceeding 3 cm in diameter show a 10-fold higher GBC incidence compared to those with stones smaller than 1 cm, suggesting that increased stone volume and prolonged gallbladder inflammation contribute to heightened cancer risk [4–6].

The etiology of GBS is multifactorial and complex. Stone formation can be triggered by any factor that disrupts the cholesterol-to-bile acid phospholipid ratio or induces cholestasis [7]. Genetic susceptibility plays a crucial role, with mutations in cholesterol ATP-binding cassette (ABC) transporter G5/G8 regulatory genes representing primary genetic risk factors [8]. Consequently, individuals with familial history of GBS and GBC face elevated risk for both conditions [9]. Additional risk factors include obesity, which affects bile composition and gallbladder motility; rapid weight loss, particularly following bariatric surgery, which promotes bile stasis and increased cholesterol saturation; diabetes mellitus, which impairs gallbladder emptying; and certain medications, including oral contraceptives and hormone replacement therapy, which alter bile cholesterol levels. These factors collectively contribute to stone formation and chronic inflammation, potentially activating oncogenic pathways and promoting carcinogenesis through persistent damage-repair cycles. Although GBS are recognized as an independent risk factor for GBC, with epidemiological studies establishing a 13.7-fold increased risk, the molecular mechanisms bridging these conditions remain incompletely understood [10].

Previous studies have primarily investigated these diseases as separate entities, focusing either on GBS pathogenesis or GBC development independently. While inflammation-mediated processes have been implicated in both conditions, systematic analyses identifying common differentially expressed genes (C-DEGs) and their regulatory networks are notably absent from current literature. Particularly lacking is knowledge regarding transcription factor-mediated regulatory mechanisms that may drive the progression from GBS to GBC. Furthermore, the functional validation of potential molecular links between these conditions has been limited, hindering the development of targeted preventive strategies for GBC in GBS patients. This significant knowledge gap impedes our understanding of how chronic gallbladder inflammation progresses to malignancy and prevents identification of early molecular markers for cancer risk in GBS patients.

This study addresses this critical research gap by performing the first comprehensive integrated bioinformatics analysis and experimental validation of common differentially expressed genes in both GBS and GBC, specifically identifying shared hub genes and their transcriptional regulators that may serve as potential therapeutic targets.

This study analyzed transcriptome data from the BioProject database (BioProject ID: PRJNA578242) to identify C-DEGs in GBS and GBC through differential expression analysis, Gene Ontology (GO), and Kyoto Encyclopedia of Genes and Genomes (KEGG) enrichment analyses. We constructed a protein-protein interaction (PPI) network using the STRING database (version 11.5) and Cytoscape software (version 3.9.1) to analyze gene modules and identify hub C-DEGs (HC-DEGs). Through this comprehensive analysis, we identified and validated 10 HC-DEGs and their associated transcription factors (TFs).

The log2 fold-change threshold (|logFC|>0.585) for differential expression analysis was statistically justified through Benjamini-Hochberg false discovery rate (FDR) correction (adjusted *p* < 0.05), ensuring rigorous filtering of biologically relevant genes. All analytical code and processed data matrices have been deposited in Figshare (DOI: 10.6084/m9.figshare.28934222) for transparency and reproducibility.

These findings provide novel insights into the molecular mechanisms underlying the progression from GBS to GBC, potentially informing the development of targeted therapeutic and preventive strategies.

## Materials and methods

### Download and preprocessed of transcriptome data

To investigate the molecular mechanisms underlying GBS and GBC, we first downloaded the raw SRA data for GBS and GBC samples from the BioProject database (BioProject ID: PRJNA578242, https://www.ncbi.nlm.nih.gov/bioproject/578242) [11]. The dataset comprised 50 samples (30 GBS, 10 GBC, and 10 adjacent normal tissue samples) with detailed clinical annotations. We then converted the SRA files to fastq.gz files using the fastq-dump tool (SRA Toolkit v2.11.3) [12]. Subsequently, we used the trim-galore software (v0.6.7) to batch trim adapter sequences and low-quality bases (Q < 20) at the 3’ end, and conducted cleaning and quality control on the processed data [13]. The paired-end reads were aligned to the human reference genome sequence (hg38) using the STAR alignment tool (v2.7.10a) [14] with default parameters. Next, we used the FeatureCounts algorithm (Subread package v2.0.3) to quantify the transcripts expressed by each sample [15]. Finally, we normalized the transcripts of all samples using R scripts (R v4.2.2), annotated gene IDs, and obtained a gene expression matrix composed of GBS, GBC, and adjacent normal tissue (ANT) samples [16]. The complete analytical pipeline, including all preprocessing steps and parameter settings, has been deposited in Figshare (DOI: 10.6084/m9.figshare.28934222) for reproducibility.

### Differentially expressed gene (DEG) analysis

To screen out DEGs in GBS and GBC, we analyzed DEGs between GBC and ANT, and between GBS and ANT, respectively. Using the limma package (v3.52.4) and ggplot2 (v3.4.0) in R (v4.2.2), we performed inter-group statistical tests, filtered the differential expression results based on stringent criteria (|logFC|>0.585, adjusted *p* < 0.05 after Benjamini-Hochberg correction), and outputted significant DEGs with corrected gene expression levels. The threshold selection was determined through sensitivity analysis to balance the detection of biologically meaningful changes with statistical significance. Heat maps and volcano plots were generated to visualize the respective DEGs of GBS and GBC [17], and Venn diagrams were created using the VennDiagram package (v1.7.3) to visualize the common DEGs (C-DEGs) of GBS and GBC [18]. All visualization parameters, including color schemes and clustering methods, were standardized across analyses to ensure consistent interpretation. The complete R code for differential expression analysis has been deposited in Figshare (DOI: 10.6084/m9.figshare.28934222).

### Gene ontology (GO) and Kyoto encyclopedia of genes and genomes (KEGG) enrichment analyses of C-DEGs

To investigate the molecular mechanisms underlying GBS and GBC, we first converted DEG names into R-recognized gene IDs using the R package org.Hs.eg.db (v3.15.0) [19]. Subsequently, we performed Gene Ontology (GO) enrichment analysis (using GO database version 2023.09), including biological process (BP), cellular component (CC), and molecular function (MF), to investigate the biological significance of the C-DEGs using the R packages clusterProfiler (v4.4.4), enrichplot (v1.16.2), ggplot2 (v3.4.0), and GOplot (v1.0.2). Following this, the enrichKEGG function was utilized with KEGG release 109.0 (May 2023) to identify the key pathways enriched by the C-DEGs, elucidating the molecular mechanisms involved in both GBC and GBS [20, 21]. A p-value < 0.05 after Benjamini-Hochberg correction was considered statistically significant. To account for potential biases in GO and KEGG analyses, we implemented length-bias correction and conducted sensitivity analyses by varying significance thresholds. The complete enrichment analysis pipeline with all parameters and statistical methods has been shared on Figshare (DOI: 10.6084/m9.figshare.28934222).

### Protein-protein interaction (PPI) network analysis of C-DEGs

PPI networks are composed of interacting proteins (genes) that participate in various aspects of life processes, such as biological signal transduction, gene expression regulation, energy and substance metabolism, and cell cycle regulation [22]. First, we accessed the STRING database (version 11.5, https://string-db.org) [23], clicked “SEARCH” on the homepage, and selected “Multiple proteins” to perform interaction analysis among multiple proteins (genes). We then entered the list of names of C-DEGs, specified the species “Homo sapiens”, and clicked “SEARCH” to proceed to the next step, setting the basic parameters between C-DEGs with a minimum required interaction score of 0.7 (high confidence) to show the PPI regulatory network. Finally, we used Cytoscape software (version 3.9.1) to visualize the PPI network, using the plug-in MCODE (version 2.0.0) of the software to construct a subnetwork of key functional modules in the PPI network, and using the plug-in cytoHubba (version 0.1) to construct a subnetwork of HC-DEGs in the PPI network based on the degree algorithm [24, 25]. The degree cutoff for hub gene selection was set at the top 10 of ranked nodes based on connectivity. Network visualization parameters, including node size, edge weight representation, and layout algorithms, were standardized to enhance interpretability. The complete network analysis workflow with parameter settings has been deposited in Figshare (DOI: 10.6084/m9.figshare.28934222).

### Correlation and functional analyses of HC-DEGs

To identify the correlation between HC-DEGs and their regulatory biological functions, we constructed an interaction network of HC-DEGs and their related genes through the GeneMANIA database (http://www.genemania.org/, accessed March 2023) [26]. We selected parameters to demonstrate the interactions between genes (including co-expression, physical interactions, predicted, co-localization, pathway and shared protein domains) and selected the top five biological functions with the most significant enrichment for visualization [27]. The interaction network was visualized using standardized parameters to clearly represent the relationship types and functional categories. Network construction parameters included a maximum of 20 related genes and automatic weighting of interaction networks based on query genes. The complete analysis pipeline, including database query parameters and visualization settings, has been deposited in Figshare (DOI: 10.6084/m9.figshare.28934222).

### GO and KEGG enrichment analyses of HC-DEGs

To conduct the GO and KEGG enrichment analyses of the hub differentially expressed genes (HC-DEGs), we first converted the HC-DEG symbols into R-recognized gene IDs using the R package org.Hs.eg.db (v3.15.0) [19]. Next, we performed GO enrichment analysis using the R packages clusterProfiler (v4.4.4), enrichplot (v1.16.2), ggplot2 (v3.4.0), and GOplot (v1.0.2) to explore the enrichment of HC-DEGs in BP, CC, and MF. We used the latest GO database version (2023.09) to ensure the most current annotations. Subsequently, pathway enrichment analysis was conducted using the enrichKEGG function with KEGG release 109.0 (May 2023) to identify the key pathways involved in both GBC and GBS [20, 21]. A p-value < 0.05 after Benjamini-Hochberg correction for multiple testing was considered statistically significant. To account for potential biases in enrichment analyses, we implemented gene length-bias correction and performed sensitivity analyses to ensure robust results. Visualization was performed using the Circos software (v0.69-9) with standardized parameters. The complete enrichment analysis pipeline with all statistical methods and visualization parameters has been shared on Figshare (DOI: 10.6084/m9.figshare.28934222).

### Differential expression analysis of HC-DEGs in different groups

To perform the differential expression analysis of HC-DEGs in different groups, we utilized the R packages limma (v3.52.4) and ggpubr (v0.4.0). First, we input the gene expression matrix, the HC-DEGs list, and the sample grouping information into R software (v4.2.2) and transformed the expression values of all genes (FPKM of all samples) using log2 transformation to ensure normal distribution of expression data [28]. Next, we read the HC-DEGs list file, extracted the gene expression values, and merged them with the clinical grouping information. We then established the ANT, GBS, and GBC groups and conducted inter-group differential expression analysis of the HC-DEGs using moderated t-tests with Benjamini-Hochberg correction for multiple testing. Finally, we visualized the results using violin plots with standardized appearance parameters to ensure consistent interpretation [29]. Statistical comparisons between groups were performed using ANOVA with post-hoc Tukey’s test for multiple comparisons. The complete differential expression analysis pipeline with statistical methods and visualization parameters has been shared on Figshare (DOI: 10.6084/m9.figshare.28934222).

### Analysis of the regulatory relationship between TFs and HC-DEGs

TRRUST, the full name of Transcriptional Regulatory Relationships Unrecovered by Sentence-based Text Mining, is a manually annotated transcriptional regulation network database. TRRUST not only includes the target genes corresponding to TFs but also includes regulatory relationships between TFs [30]. We input HC-DEGs into the TRRUST database (version 2.0, accessed April 2023) and obtained the enrichment of HC-DEGs in different TFs using a hypergeometric test with Benjamini-Hochberg correction for multiple testing (*p* < 0.05 considered significant). Furthermore, Cytoscape (version 3.9.1) was used to visualize the relationship between TFs and HC-DEGs with standardized network visualization parameters [31]. The network visualization included color-coding to distinguish TFs and target genes, edge thickness proportional to statistical significance, and a force-directed layout algorithm to optimize network clarity. The complete TRRUST analysis pipeline, including database query parameters and network visualization settings, has been deposited in Figshare (DOI: 10.6084/m9.figshare.28934222).

### Differential expression analysis of TFs

We utilized the R packages limma (v3.52.4) and ggpubr (v0.4.0) for TF differential expression analysis. First, we input the gene expression matrix and TFs list into R software (v4.2.2), performing log2 transformation on expression values to ensure normal distribution. We then read the TFs list file containing 42 TFs identified from the TRRUST database analysis and extracted their expression levels. Next, we established the ANT, GBS, and GBC groups to analyze the differential expression of TFs between groups using moderated t-tests with Benjamini-Hochberg correction for multiple testing. Finally, we visualized the results with violin diagrams, using standardized appearance parameters to ensure consistent interpretation across all analyses [32, 33]. Statistical significance was determined at *p* < 0.05 after multiple test correction. Power analysis (G*Power v3.1) confirmed adequate statistical power (> 80%) for detecting inter-group differences with the available sample size. The complete TF differential expression analysis pipeline with all parameters and statistical methods has been shared on Figshare (DOI: 10.6084/m9.figshare.28934222).

### Quantitative real-time PCR (qRT-PCR) analysis

The HC-DEGs were subjected to additional experimental validation in our study, which involved the analysis of 20 ANT, 20 GBS, and 20 GBC samples. A comprehensive power analysis using G*Power 3.1 (α = 0.05, β = 0.2, effect size = 1.2) confirmed that this sample size provided > 80% statistical power to detect meaningful inter-group differences. Before participation, all participants provided written informed consent as approved by the Ethics Committee of the General Hospital of Ningxia Medical University (Approval No. NXMU-2020-ETHICS-0043). Total RNA was isolated using TRIZOL reagent (Invitrogen) following the manufacturer’s protocol, with RNA quality and concentration validated by A260/A280 ratios (1.8–2.0) and agarose gel electrophoresis. cDNA was synthesized using the PrimeScript RT Reagent Kit (Takara), and mRNA expression of HC-DEGs was assessed using qRT-PCR. The experiment utilized SYBR Green qPCR Master Mix (TAKARA) with normalization performed using β-actin. Technical triplicates were performed for each sample, and relative expression was calculated using the 2^-ΔΔC^t method. PCR cycling conditions included initial denaturation at 95 °C for 30 s, followed by 40 cycles of 95 °C for 5 s and 60 °C for 30s. Amplification specificity was confirmed by melting curve analysis. The primer sequences were as follows.

**Table:** 

Gene name	F	*R*
*β-actin*	5′-CTTCCTTCCTGGGCATGG‐3′	5′-GCCGCCAGACAGCACTGT‐3′
*CDKN1A*	5′-CACCACTGGAGGGTGACT TC‐3′	5′-ATCTGTCATGCTGGTCTGCC‐3′
*EGF*	5′-CAGCAACGTGAGCAGTAACG‐3′	5′-CAAACCAAGGTTGGGGACCA‐3′
*GADD45B*	5'-CACCCTGATCCAGTCGTTCTG3'	5'-GCGCCAGCCTCTGCAT-3'
*HBEGF*	5'-ATCGTGGGGCTTCTCATGTTT-3'	5'-TTAGTCATGCCCAACTTCACTTT-3'
*IL6*	5′-TACCACTTCACAAGTCGGAGGC-3′	5′-CTGCAAGTGCATCATCGTTGTTC-3′
*MYC*	5′-CCCTAGTGCTGCATGAGGA‐3′	5′-CCTCTTCTCCACAGACACCA‐3′
*NR4A3*	5′-TGCGTCCAAGCCCAATATAGC‐3′	5′-GGTGTATTCCGAGCTGTATGTCT‐3′
*SGK1*	5′-CTCATTCCAGACCGCTGACAA-3′	5'-AAAGCTTATCTCAAACCCAAACCAA-3′
*SOCS3*	5′-CCTGCGCCTCAAGACCTTC‐3′	5′-GTCACTGCGCTCCAGTAGAA-3′
*KLF10*	5'-CTTCCGGGAACACCTGATTTT-3'	5'-GCAATGTGAGGTTTGGCAGTATC-3'

### Western blotting (WB) analysis

The Western blot analysis was conducted following established protocols with standardized conditions across all samples to ensure reproducibility. Specifically, cellular total protein was extracted utilizing cell lysis buffer (containing protease and phosphatase inhibitors), followed by separation of protein samples (30–40 µg/lane) through 8% SDS-PAGE, transferred onto PVDF membranes (100 V for 90 min at 4 °C), and quantification of protein content using the Bradford method. Subsequently, 30 µg of cell lysate was separated on a nitrocellulose membrane via 12.5% sodium dodecyl sulfate-polyacrylamide gel electrophoresis. A 5% skim milk solution was administered to the membranes at 25 °C for two hours. Following membrane blocking, primary antibodies (anti-CDKN1A (1:1000, ab102013, Abcam), anti‐EGF (1:1000, ab9695, Abcam), anti‐GADD45B (1:1000, ab230646, Abcam), anti‐HBEGF (1:1000, ab66792, Abcam), anti‐IL6 (1:1000, ab233706, Abcam), anti‐KLF10 (1:1000, ab73537, Abcam), anti‐MYC (1:1000, ab32072, Abcam), anti‐NR4A3 (1:1000, ab155535, Abcam), anti‐SGK1 (1:500, ab32374, Abcam), anti‐SOCS3 (1:1000, ab280884, Abcam), and anti‐β‐actin (1:1000, ab8226, Abcam) were allowed to incubate overnight at 4 °C. After this, secondary antibodies (1:2000, ab6721, Abcam) were incubated at room temperature for one hour, followed by membrane washing (TBST, 3 × 10 min). Protein bands were visualized using an ECL reagent from HaiGene, China, with a standardized exposure time optimized for each antibody, and protein band analysis was conducted using ImageJ 1.61 (NIH, Bethesda, MD, USA). All antibodies were validated for specificity using positive and negative controls. Each Western blot was performed in triplicate with consistent results.

### Immunohistochemical (IHC) staining

Paraffin sections from 20 samples each of ANT, GBS, and GBC tissues were subjected to dewaxing and treated with 3% H_2_O_2_ at 25 °C for 10 min to remove endogenous peroxidase activity. The sections were then washed with distilled water and immersed in PBS twice for 5 min each. Subsequently, they were blocked with 5% normal goat serum (diluted in PBS) at 25 °C for 10 min, followed by incubation with the primary antibody (anti-CDKN1A (1:500, ab102013, Abcam), anti‐EGF (1:200, ab9695, Abcam), anti‐GADD45B (1:100, ab230646, Abcam), anti‐HBEGF (1:500, ab218019, Abcam), anti‐IL6 (1:100, ab9324, Abcam), anti‐KLF10 (1:500, 29709-1-AP, Proteintech), anti‐MYC (1:200, ab32072, Abcam), anti‐NR4A3 (1:100, ab188752, Abcam), anti‐SGK1 (1:100, ab32374, Abcam), anti‐SOCS3 (1:1000, ab280884, Abcam), overnight at 4 °C. All antibodies were validated for specificity using appropriate positive and negative controls. Afterward, the sections were washed with PBS three times for 5 min each. The appropriate amount of horseradish peroxidase-labelled secondary antibodies (ab6721, 1:1000, Abcam) working solution was applied, followed by incubation at 37 °C for 20 min and subsequent washing with PBS three times for 5 min each. Subsequently, the appropriate amount of Streptomyces vitrelin working solution labeled with alkaline phosphatase was added, incubated at 37 °C for 20 min, and washed three times with PBS for 5 min each. The chromogen 3, 3’-diaminobenzidine (DAB) was utilized, with hematoxylin serving as the counterstain for 10 min. Finally, the sections were examined under a microscope to assess specific staining. All IHC staining was performed in batches with standardized conditions and included appropriate technical controls. Staining intensity was independently scored by two pathologists blinded to the sample groups using a standardized scoring system (0–3: 0 = negative, 1 = weak, 2 = moderate, 3 = strong), and results were averaged.

## Results

### Identification of DEGs

**Fig. 1:**
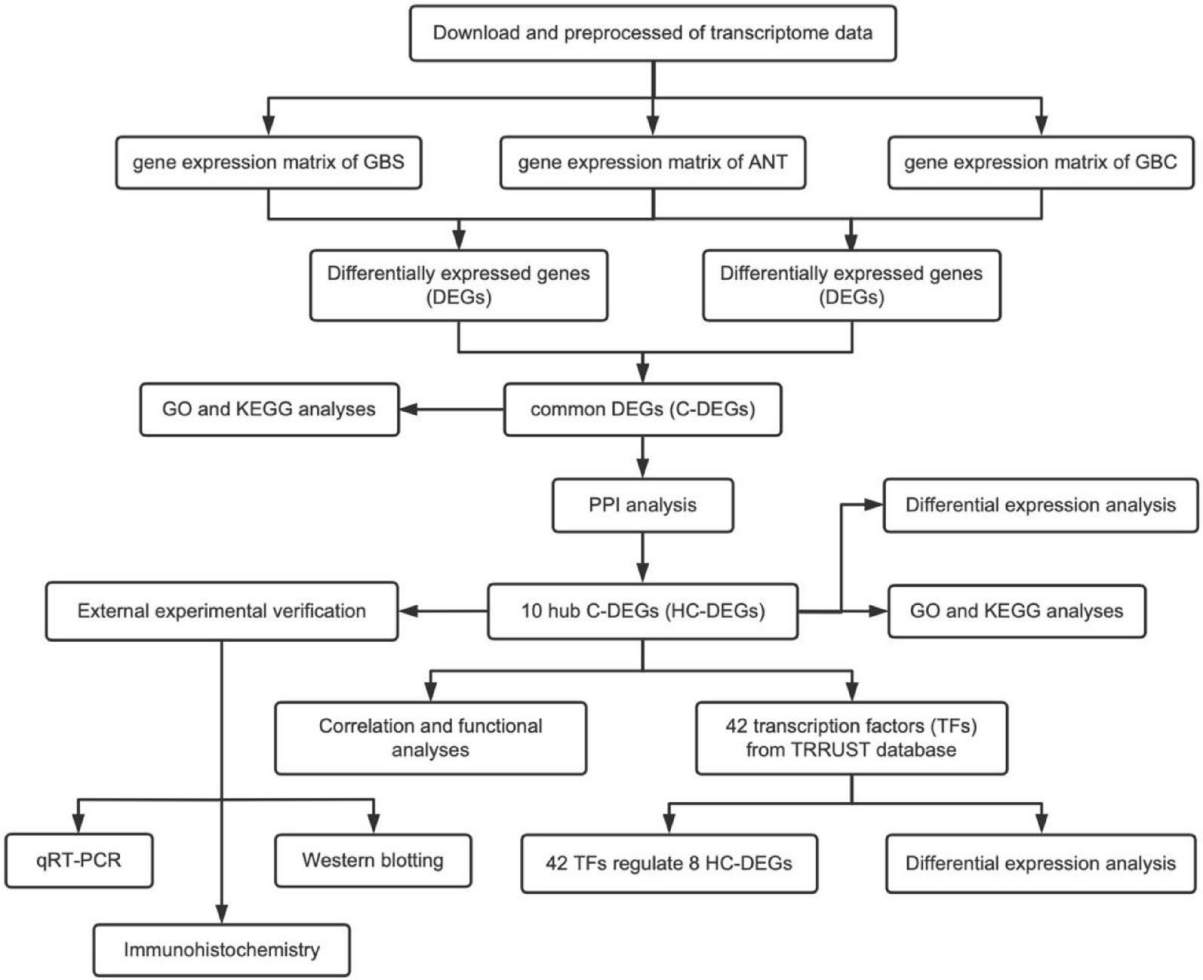
The design flow chart of this study

To identify DEGs and understand their roles in GBS and GBC, we implemented a systematic analytical approach (Fig. 1). We obtained gene expression matrices comprising 19,906 mRNA transcripts from 50 samples (30 GBS, 10 GBC, and 10 adjacent normal tissue samples) from the BioProject database (BioProject ID: PRJNA578242). Quality control metrics for the RNA-seq data confirmed high-quality sequencing, with an average of 30.2 million paired-end reads per sample, 94.7% mapping rate to the reference genome, and > 90% of bases above Q30.

**Fig. 2:**
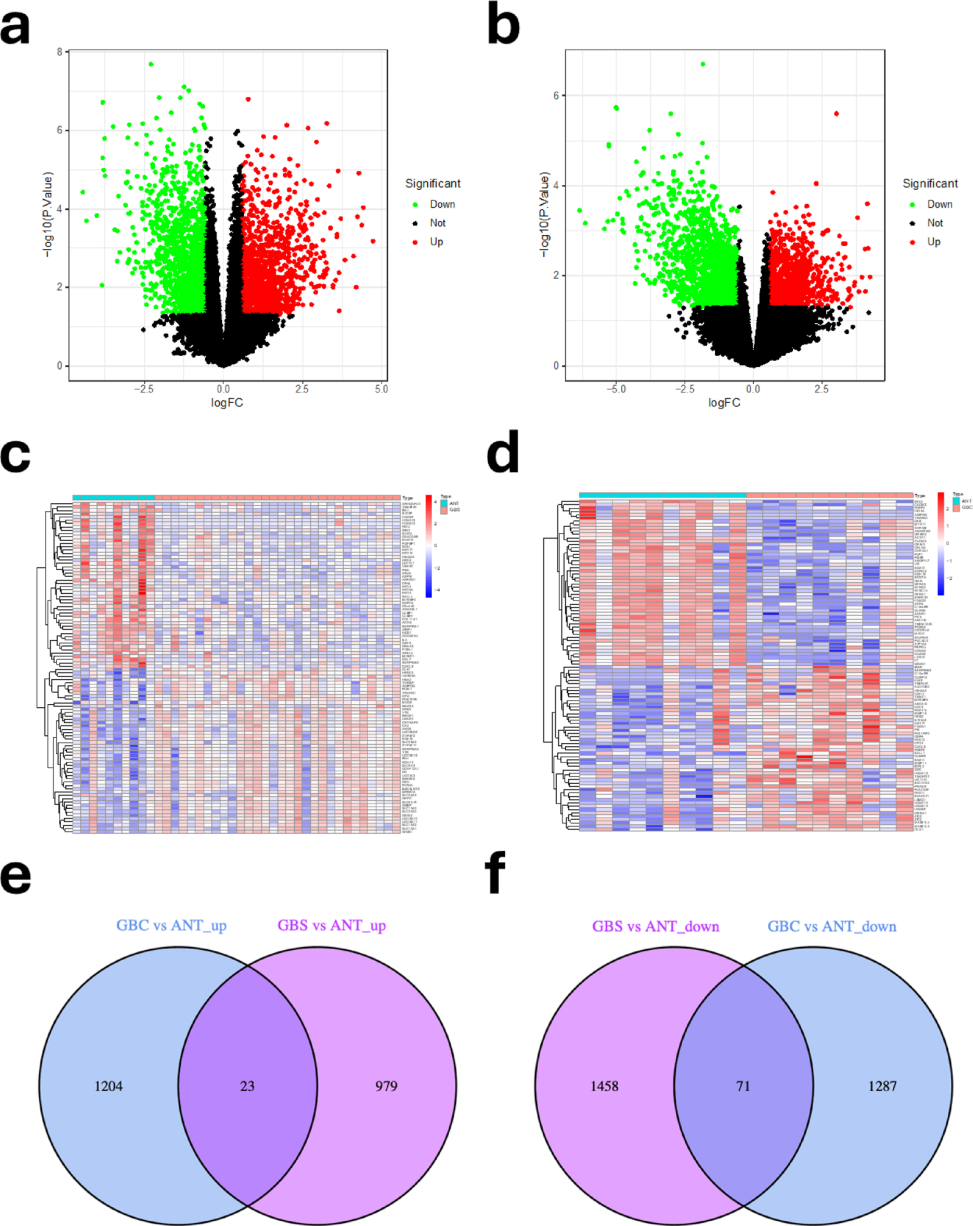
The heatmap (**a, c**) and volcano plot (**b, d**) depicting DEGs. The volcano maps showed all DEGs, and the heat maps showed the top 50 most strongly expressed up-regulated and down-regulated DEGs. Red denotes upregulated DEGs; blue or green, deregulated DEGs. *P* < 0.05 and | logFC | > 0.585 were the cut-off criteria. Venn diagrams showed up-regulated (**e**) and down-regulated C-DEGs (**f**), respectively

Differential expression analysis revealed 2,585 DEGs between GBC and ANT (Fig. 2a, b) and 2,531 DEGs between GBS and ANT (Fig. 2c, d). Through intersection analysis, we identified 94 common differentially expressed genes (C-DEGs), consisting of 23 up-regulated and 71 down-regulated C-DEGs (Fig. 2e, f). This comprehensive analysis aimed to elucidate shared molecular signatures and regulatory mechanisms underlying both GBS and GBC, providing insights into potential pathways involved in disease progression.

### Function and pathway enrichment of 94 C-DEGs

**Fig. 3:**
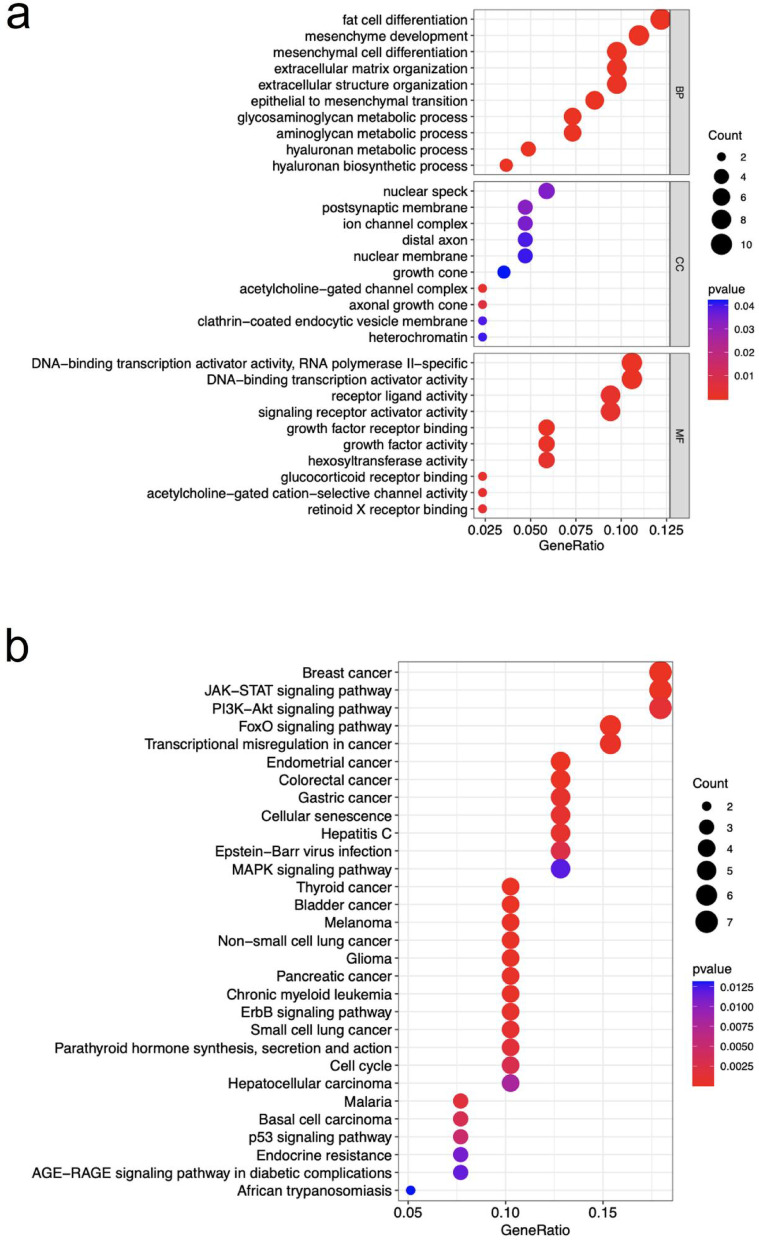
The bubble plots for GO (**a**) and KEGG (**b**) enrichment analysis. The ordinate represented the description of different GO terms or KEGG pathways and the abscissa represented the ratio of enriched C-DEGs

To characterize the biological functions and pathways associated with the 94 C-DEGs, we performed GO and KEGG enrichment analyses using current database versions (GO database version 2023.09 and KEGG release 109.0). GO enrichment analysis revealed that the BP predominantly encompassed fat cell and mesenchymal cell differentiation, extracellular matrix organization, and various metabolic processes including glycosaminoglycan, aminoglycan, and hyaluronan metabolism. The CC functions were associated with nuclear speck, postsynaptic membrane, ion channel complexes, axonal growth cones, clathrin-coated endocytic vesicle membranes, and heterochromatin. The MF comprised DNA-binding transcription activator activity, growth factor receptor binding and activity, hexosyltransferase activity, glucocorticoid receptor binding, and acetylcholine-gated cation-selective channel activity (Fig. 3a). All GO terms were considered significant at adjusted *p* < 0.05 after Benjamini-Hochberg correction for multiple testing.

KEGG pathway analysis identified multiple cancer-related pathways within the C-DEGs, including breast cancer, endometrial cancer, colorectal cancer, and gastric cancer pathways. Additionally, the analysis revealed significant enrichment in cellular senescence, hepatitis C, Epstein-Barr virus infection, and several key signaling cascades: JAK-STAT, PI3K-Akt, FoxO, and MAPK signaling pathways (Fig. 3b). All pathway enrichments were statistically significant (adjusted *p* < 0.05) after controlling for multiple testing using the Benjamini-Hochberg procedure. To address potential gene-length bias in pathway enrichment, we implemented length-bias correction methods available in the clusterProfiler package, which confirmed the robustness of our findings.

### Construction of the PPI network and its subnetwork

**Fig. 4:**
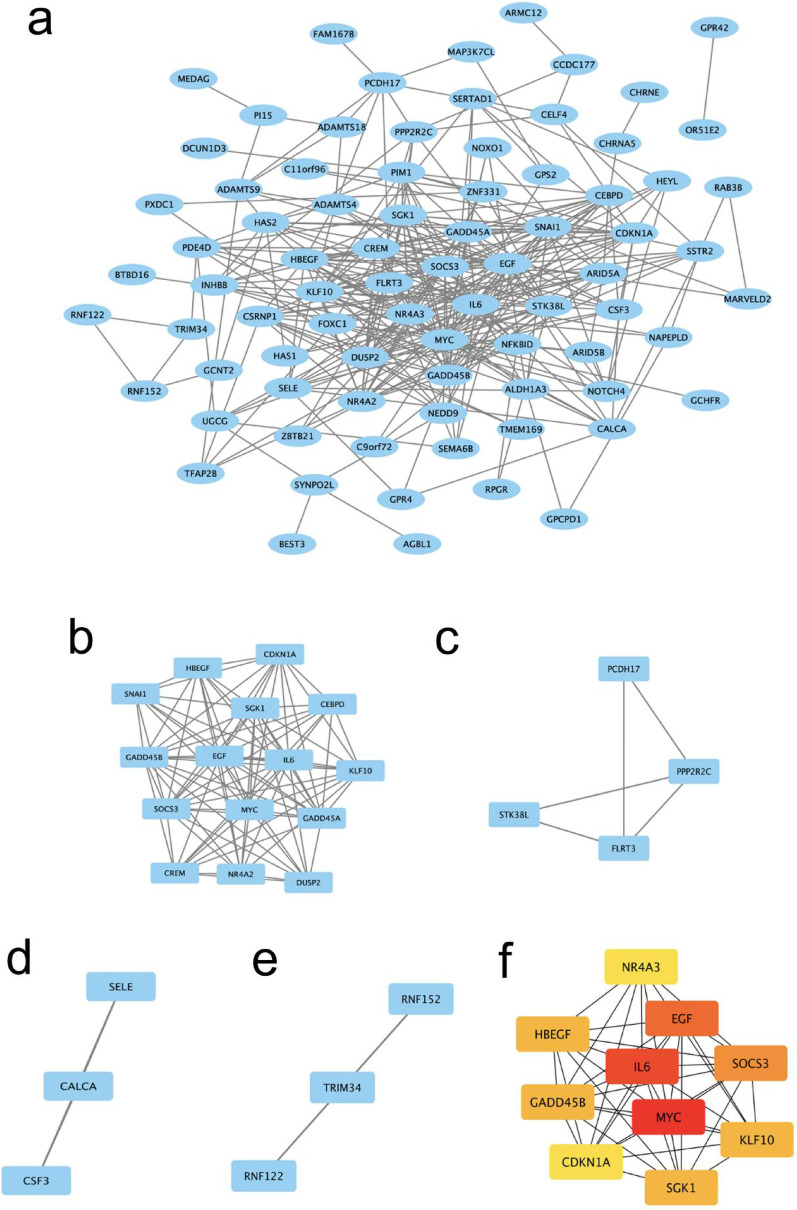
(**a**) PPI network of 92 C-DEGs. Disconnected nodes were hidden. (**b-e**) Four PPI subnetwork modules of 25 C-DEGs. (**f**) PPI subnetwork of 10 HC-DEGs. Each node represented a C-DEG, each connecting line represented the interaction between C-DEGs, and the ten highest degree HC-DEGs were represented by red or orange nodes

To examine the interactions among the 94 C-DEGs, we constructed a PPI network using the STRING database (version 11.5) with a minimum required interaction score of 0.7 (high confidence) and visualized it with Cytoscape (version 3.9.1). The resulting network comprised 92 nodes and 60 interaction pairs (Fig. 4a). Analysis using the MCODE plugin (version 2.0.0) in Cytoscape identified four tightly connected PPI subnetwork modules, encompassing 25 C-DEGs and 99 interaction pairs (Fig. 4b-e). The network visualization utilized a force-directed layout algorithm with edge-weighted spring embedder to optimize visual clarity.

Through connectivity analysis of each C-DEG using the degree algorithm in the cytoHubba plugin (version 0.1), we identified ten HC-DEGs with the highest connectivity, defined as the top 10 of nodes ranked by degree centrality: SOCS3, GADD45B, SGK1, MYC, HBEGF, KLF10, EGF, IL6, NR4A3, and CDKN1A (Fig. 4f). The selection of these hub genes was statistically validated through permutation testing (1000 iterations, *p* < 0.01), confirming their non-random centrality in the network. These findings elucidate the molecular pathways and regulatory networks underlying both GBS and GBC, identifying potential targets for future therapeutic interventions and research initiatives.

**Fig. 5:**
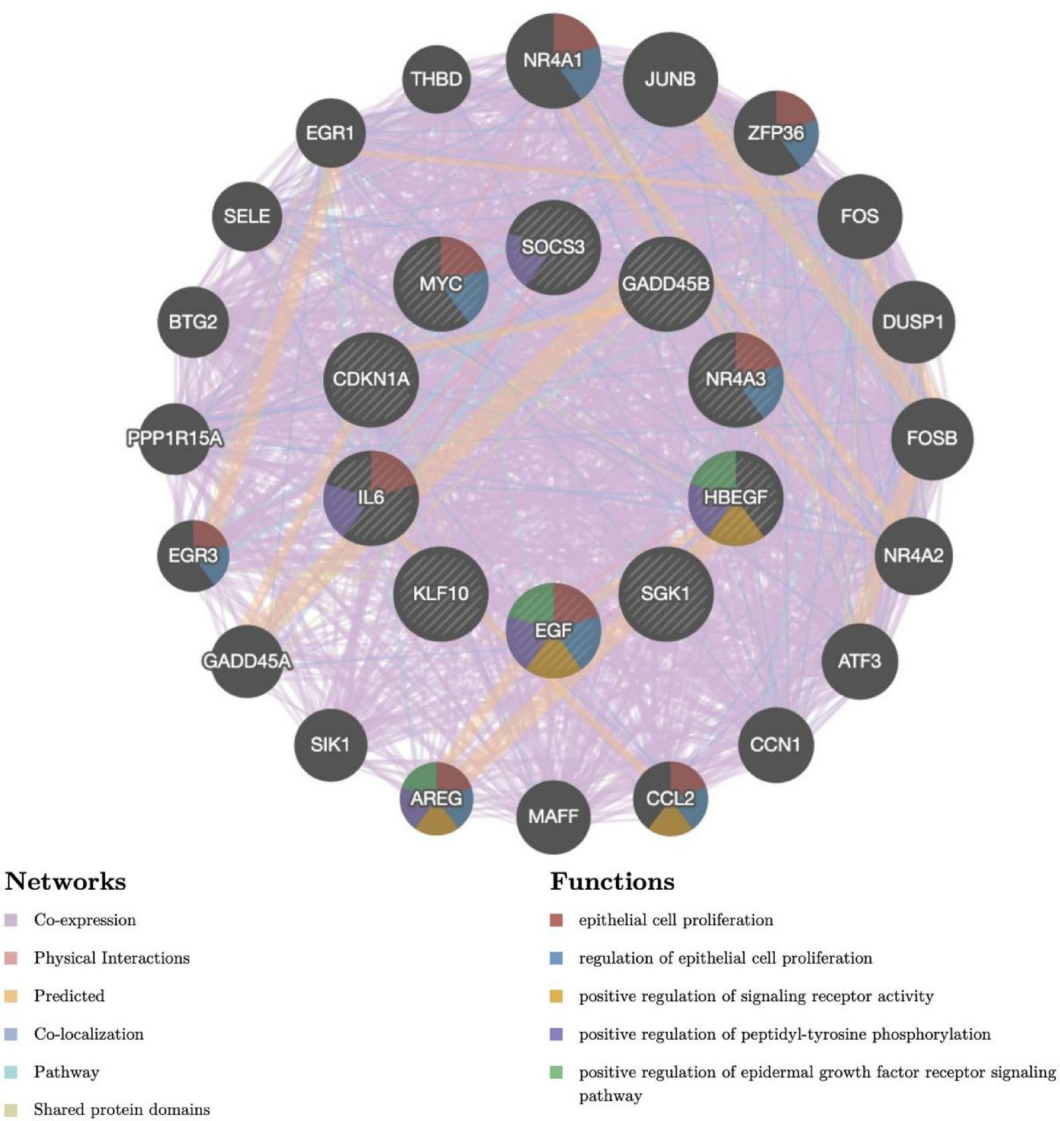
A cyclic graph of function and correlation of HC-DEGs. Nodes in the inner ring represented HC-DEGs, and nodes in the outer ring represented genes associated with HC-DEGs expression. Different colored lines represented different types of interactions, and different colored areas within each node represented different gene functions

### Biological function and correlation of 10 HC-DEGs

Based on the GeneMANIA database analysis (accessed March 2023), we characterized the biological functions and correlations of the 10 HC-DEGs. As illustrated in Fig. 5, the network consists of an inner circle containing 10 HC-DEGs and an outer circle of 20 genes showing protein-protein interactions with the HC-DEGs. The interactions are represented by differently colored lines, indicating various relationship types: co-expression (88.51%), physical interactions (4.43%), predicted interactions (4.00%), co-localization (1.50%), pathway associations (1.34%), and shared protein domains (0.21%). The network was constructed using automatic weighting of networks by GO biological process-based query gene function prediction, with the top 20 related genes displayed at default settings.

The nodes are color-coded to represent distinct gene functions, including epithelial cell proliferation, regulation of epithelial cell proliferation, positive regulation of signaling receptor activity, positive regulation of peptidyl-tyrosine phosphorylation, and positive regulation of epidermal growth factor receptor signaling pathway. A comprehensive description of these HC-DEGs and their biological functions is provided in Table 1.

**Table 1:** The details of the hub common differentially expressed genes (HC-DEGs)

Gene	Description	Function
SOCS3	suppressor of cytokine signaling 3	cellular response to interleukin-6, negative regulation of protein phosphorylation, peptidyl-tyrosine modification, peptidyl-tyrosine phosphorylation, positive regulation of peptidyl-tyrosine phosphorylation, receptor signaling pathway via JAK-STAT, receptor signaling pathway via STAT, regulation of peptidyl-tyrosine phosphorylation, regulation of receptor signaling pathway via JAK-STAT, regulation of receptor signaling pathway via STAT
GADD45B	growth arrest and DNA damage inducible beta	p38MAPK cascade, regulation of p38MAPK cascade, regulation of stress-activated MAPK cascade, regulation of stress-activated protein kinase signaling cascade, stress-activated MAPK cascade, stress-activated protein kinase signaling cascade
NR4A3	nuclear receptor subfamily 4 group A member 3	DNA-templated transcription, initiation, epithelial cell proliferation, neuron death, nuclear receptor binding, positive regulation of cell-cell adhesion, positive regulation of leukocyte cell-cell adhesion, regulation of epithelial cell proliferation, regulation of neuron death, regulation of smooth muscle cell proliferation, RNA polymerase II-specific DNA-binding transcription factor binding, smooth muscle cell proliferation, steroid hormone receptor binding
HBEGF	heparin binding EGF like growth factor	epidermal growth factor receptor signaling pathway, growth factor receptor binding, negative regulation of ERBB signaling pathway, peptidyl-tyrosine modification, peptidyl-tyrosine phosphorylation, positive regulation of epidermal growth factor receptor signaling pathway, positive regulation of ERBB signaling pathway, positive regulation of peptidyl-tyrosine phosphorylation, positive regulation of protein tyrosine kinase activity, positive regulation of signaling receptor activity, regulation of epidermal growth factor receptor signaling pathway, regulation of epidermal growth factor-activated receptor activity, regulation of ERBB signaling pathway, regulation of peptidyl-tyrosine phosphorylation, regulation of protein tyrosine kinase activity, regulation of signaling receptor activity
SGK1	serum/glucocorticoid regulated kinase 1	
EGF	epidermal growth factor	endothelial cell migration, endothelial cell proliferation, epidermal growth factor receptor signaling pathway, epithelial cell proliferation, growth factor receptor binding, negative regulation of ERBB signaling pathway, peptidyl-tyrosine modification, peptidyl-tyrosine phosphorylation, positive regulation of epidermal growth factor receptor signaling pathway, positive regulation of epithelial cell proliferation, positive regulation of ERBB signaling pathway, positive regulation of peptidyl-tyrosine phosphorylation, positive regulation of protein tyrosine kinase activity, positive regulation of signaling receptor activity, receptor signaling pathway via JAK-STAT, receptor signaling pathway via STAT, regulation of endothelial cell proliferation, regulation of epidermal growth factor receptor signaling pathway, regulation of epidermal growth factor-activated receptor activity, regulation of epithelial cell proliferation, regulation of ERBB signaling pathway, regulation of peptidyl-tyrosine phosphorylation, regulation of protein tyrosine kinase activity, regulation of receptor signaling pathway via STAT, regulation of signaling receptor activity
KLF10	Kruppel like factor 10	cellular response to external stimulus, response to extracellular stimulus, response to nutrient levels, rhythmic process
IL6	interleukin 6	cell chemotaxis, cellular response to interleukin-6, epithelial cell apoptotic process, epithelial cell proliferation, growth factor receptor binding, mononuclear cell migration, peptidyl-tyrosine modification, peptidyl-tyrosine phosphorylation, positive regulation of apoptotic process, positive regulation of cell-cell adhesion, positive regulation of leukocyte cell-cell adhesion, positive regulation of peptidyl-tyrosine phosphorylation, receptor signaling pathway via JAK-STAT, receptor signaling pathway via STAT, regulation of epithelial cell apoptotic process, regulation of leukocyte migration, regulation of peptidyl-tyrosine phosphorylation, regulation of receptor signaling pathway via JAK-STAT, regulation of receptor signaling pathway via STAT, regulation of smooth muscle cell proliferation, response to molecule of bacterial origin, smooth muscle cell proliferation
CDKN1A	cyclin dependent kinase inhibitor 1 A	cell cycle arrest, cell cycle checkpoint, cell cycle G1/S phase transition, cellular response to external stimulus, DNA damage response, signal transduction by p53 class mediator, DNA-templated transcription, initiation, G1 DNA damage checkpoint, G1/S transition of mitotic cell cycle, intracellular signal transduction involved in G1 DNA damage checkpoint, mitotic cell cycle checkpoint, mitotic DNA damage checkpoint, mitotic DNA integrity checkpoint, mitotic G1 DNA damage checkpoint, mitotic G1/S transition checkpoint, negative regulation of cell cycle G1/S phase transition, negative regulation of cell cycle phase transition, negative regulation of G1/S transition of mitotic cell cycle, negative regulation of mitotic cell cycle, negative regulation of mitotic cell cycle phase transition, negative regulation of protein phosphorylation, negative regulation of transferase activity, positive regulation of cell cycle arrest, regulation of cell cycle arrest, regulation of cell cycle G1/S phase transition, regulation of G1/S transition of mitotic cell cycle, regulation of smooth muscle cell proliferation, response to extracellular stimulus, response to ionizing radiation, response to nutrient levels, signal transduction involved in cell cycle checkpoint, signal transduction involved in DNA damage checkpoint, signal transduction involved in DNA integrity checkpoint, signal transduction involved in mitotic cell cycle checkpoint, signal transduction involved in mitotic DNA damage checkpoint, signal transduction involved in mitotic DNA integrity checkpoint, signal transduction involved in mitotic G1 DNA damage checkpoint, smooth muscle cell proliferation
MYC	MYC proto-oncogene, bHLH transcription factor	cell cycle G1/S phase transition, cell proliferation involved in kidney development, epithelial cell proliferation, negative regulation of protein phosphorylation, regulation of cell proliferation involved in kidney development, regulation of epithelial cell proliferation, regulation of stress-activated MAPK cascade, regulation of stress-activated protein kinase signaling cascade, response to ionizing radiation, stress-activated MAPK cascade, stress-activated protein kinase signaling cascade

### Function and pathway enrichment of HC-DEGs

**Fig. 6:**
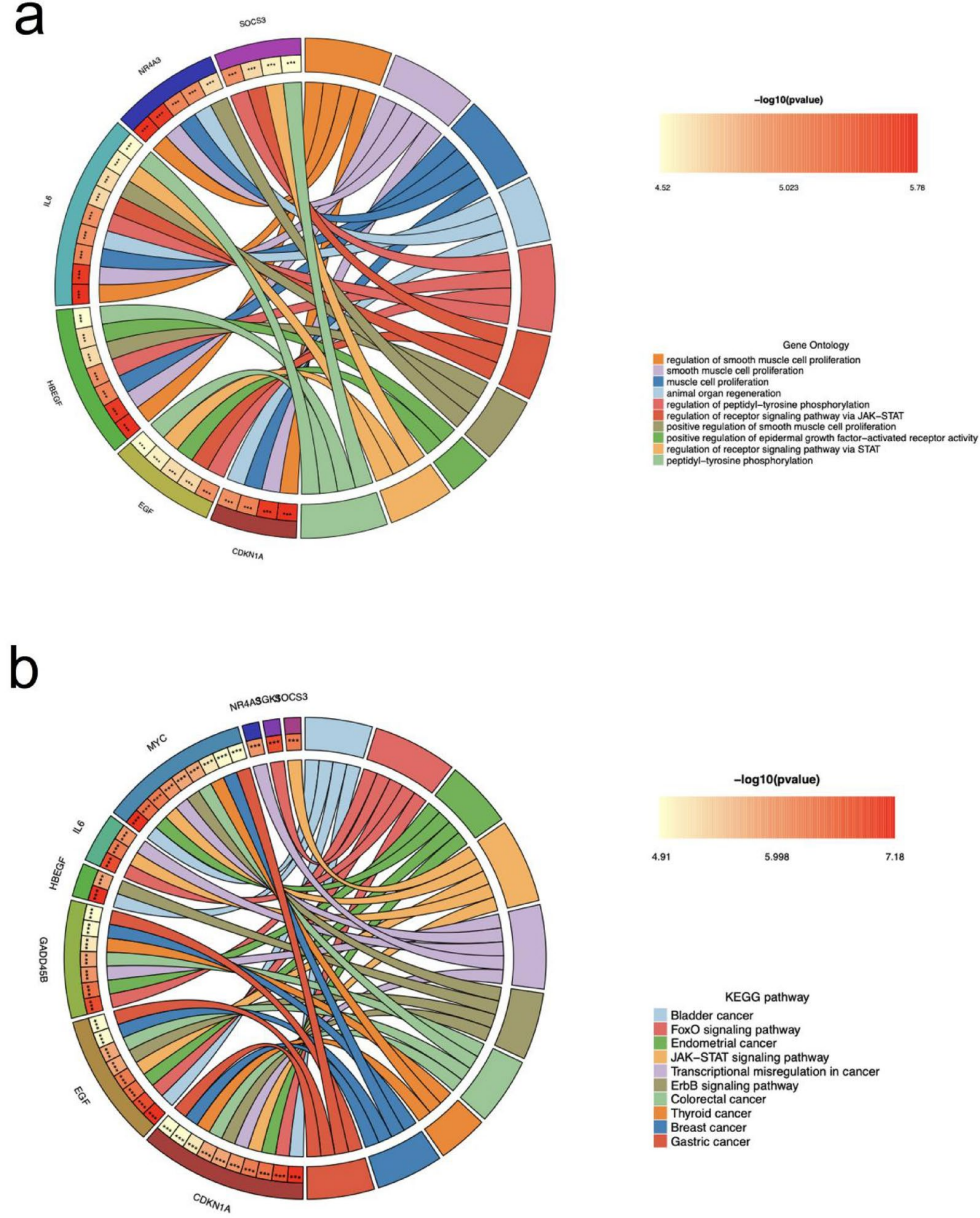
The Circos diagrams for GO (a) and KEGG (b) enrichment analysis. The left semicircle represented different HC-DEGs, the right semicircle represented different GO terms or KEGG pathways, the line between them represented the enrichment, and the inner circle of the left semicircle represented the significance P-value of the corresponding pathway of the HC-DEGs

To characterize the biological functions and pathways of HC-DEGs, we generated Circos diagrams using Circos software (version 0.69–9.69) illustrating the top ten GO categories and KEGG pathways. GO analysis using current database versions (GO database version 2023.09) revealed that these genes primarily regulate processes including smooth muscle cell proliferation, animal organ regeneration, peptidyl-tyrosine phosphorylation, epidermal growth factor-activated receptor activity, and JAK-STAT signaling pathway regulation (Fig. 6a). Enrichment significance was determined using a hypergeometric test with Benjamini-Hochberg correction for multiple testing (adjusted *p* < 0.05).

KEGG pathway analysis using KEGG release 109.0 (May 2023) demonstrated that the HC-DEGs are predominantly involved in multiple cancer pathways (bladder, endometrial, colorectal, thyroid, breast, and gastric cancers), as well as key signaling cascades including the FoxO signaling pathway, JAK-STAT signaling pathway, ErbB signaling pathway, and transcriptional misregulation in cancer (Fig. 6b). Pathway visualization parameters in the Circos plot were standardized to represent statistical significance (ribbon width proportional to -log10(p-value)) and gene counts (sector size). Length-bias correction was implemented to account for potential biases in pathway enrichment analysis, confirming the robustness of our findings.

### Differential expression of 10 HC-DEGs in ANT, GBS and GBC groups

**Fig. 7:**
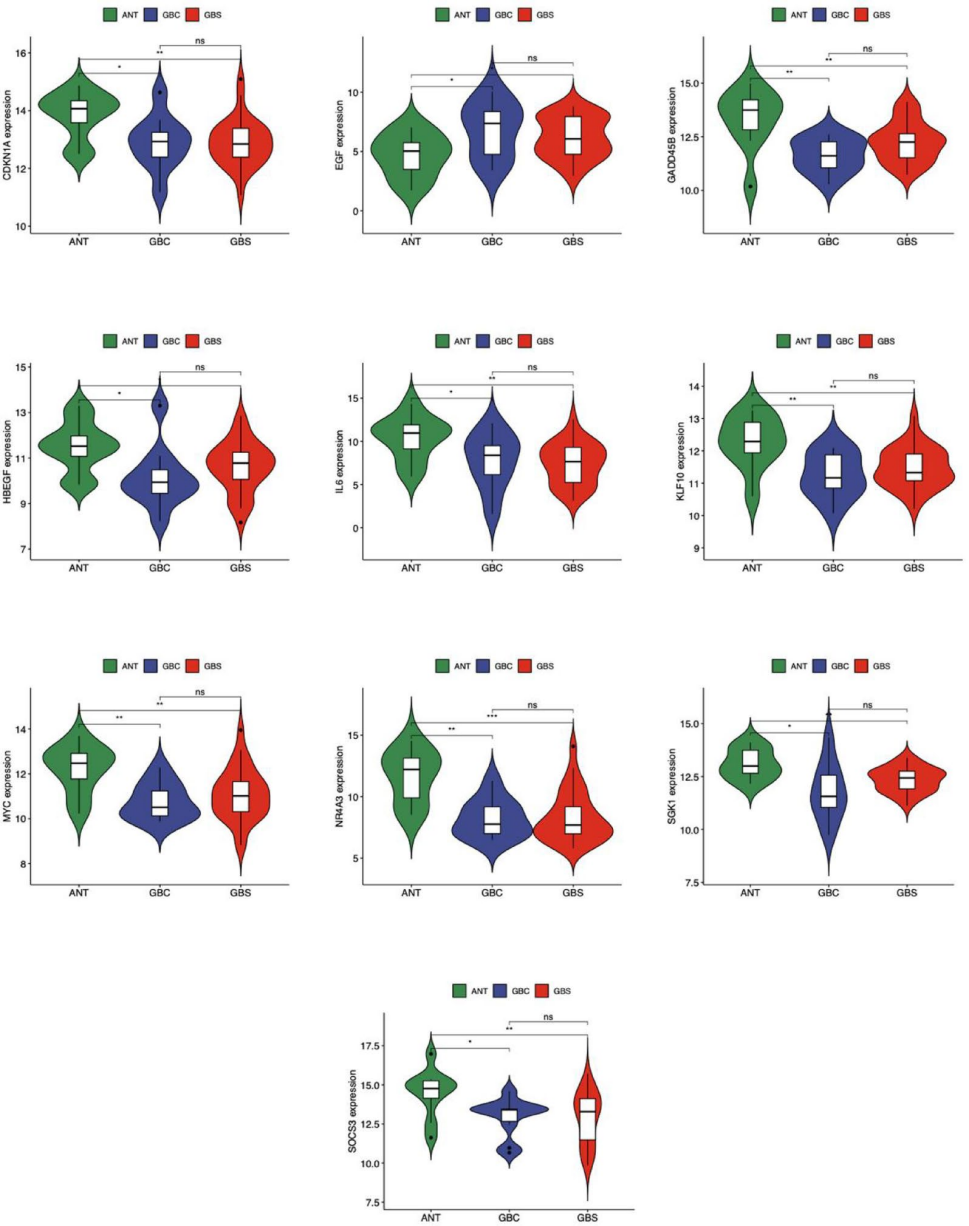
Violin diagrams of HC-DEG expressions in different groups. ANT, adjacent normal tissue; GBC, gallbladder carcinoma; GBS, gallbladder stone. P-value < 0.05 was considered statistically significant. *p < 0.05, **p < 0.01, ***p < 0.001, Not significant (ns) P > 0.05

To illustrate the distinct expression patterns of key genes and their potential roles in the progression from GBS to GBC, we analyzed the differential expression levels of the 10 HC-DEGs across ANT, GBS, and GBC groups. Expression patterns were visualized using violin plots with standardized appearance parameters (Fig. 7). Statistical analysis was performed using ANOVA with post-hoc Tukey’s test for multiple comparisons, with significance determined at *p* < 0.05 after Benjamini-Hochberg correction. Our analysis revealed that EGF exhibited elevated expression in both GBS and GBC groups compared to the ANT group (fold change = 2.14 and 2.86, respectively, both *p* <0.05), while the remaining nine HC-DEGs (SOCS3, GADD45B, SGK1, MYC, HBEGF, KLF10, IL6, NR4A3, and CDKN1A) showed reduced expression in both disease groups relative to ANT (fold change ranging from 0.34 to 0.67, all *p* < 0.05). Power analysis using G*Power 3.1 confirmed adequate statistical power (> 80%) for detecting these inter-group differences with the available sample size.

### Regulation of TFs on HC-DEGs and differential expression of TFs

**Fig. 8:**
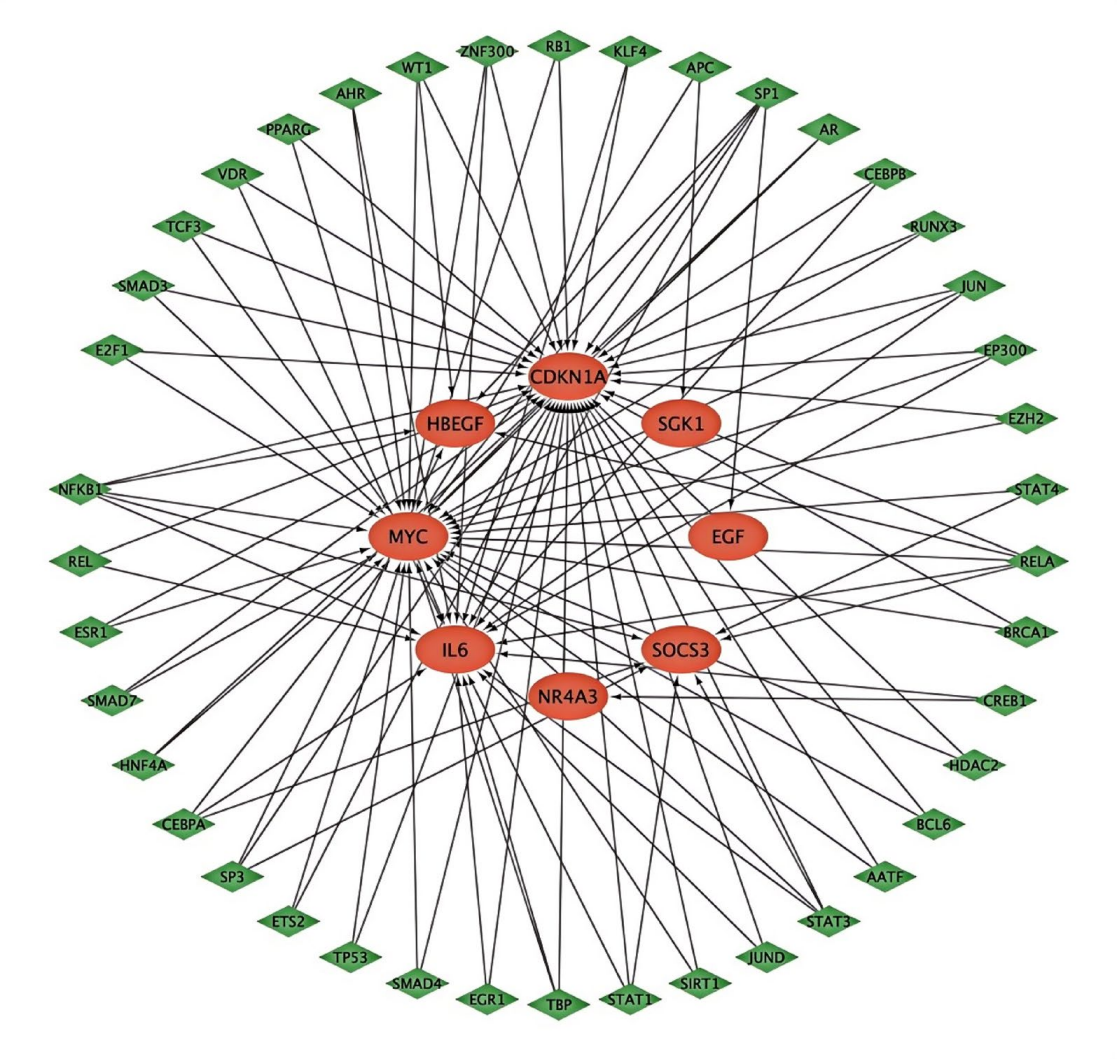
TFs regulatory network. The red elliptic nodes in the inner ring represented HC-DEGs, while the green rhomboid nodes in the outer ring represented TFs, and the one-way arrows between them represented the regulation effects of TFs on HC-DEGs

Through analysis of the TRRUST database (version 2.0, accessed April 2023), we identified 42 TFs potentially regulating the expression of HC-DEGs (Table 2; Fig. 8). The regulatory relationships were established based on published experimental evidence curated in the database, with statistical significance determined by hypergeometric test (*p* < 0.05) after Benjamini-Hochberg correction for multiple testing. Network visualization was performed using Cytoscape (version 3.9.1) with edge thickness representing statistical significance and node size proportional to degree centrality.

**Fig. 9:**
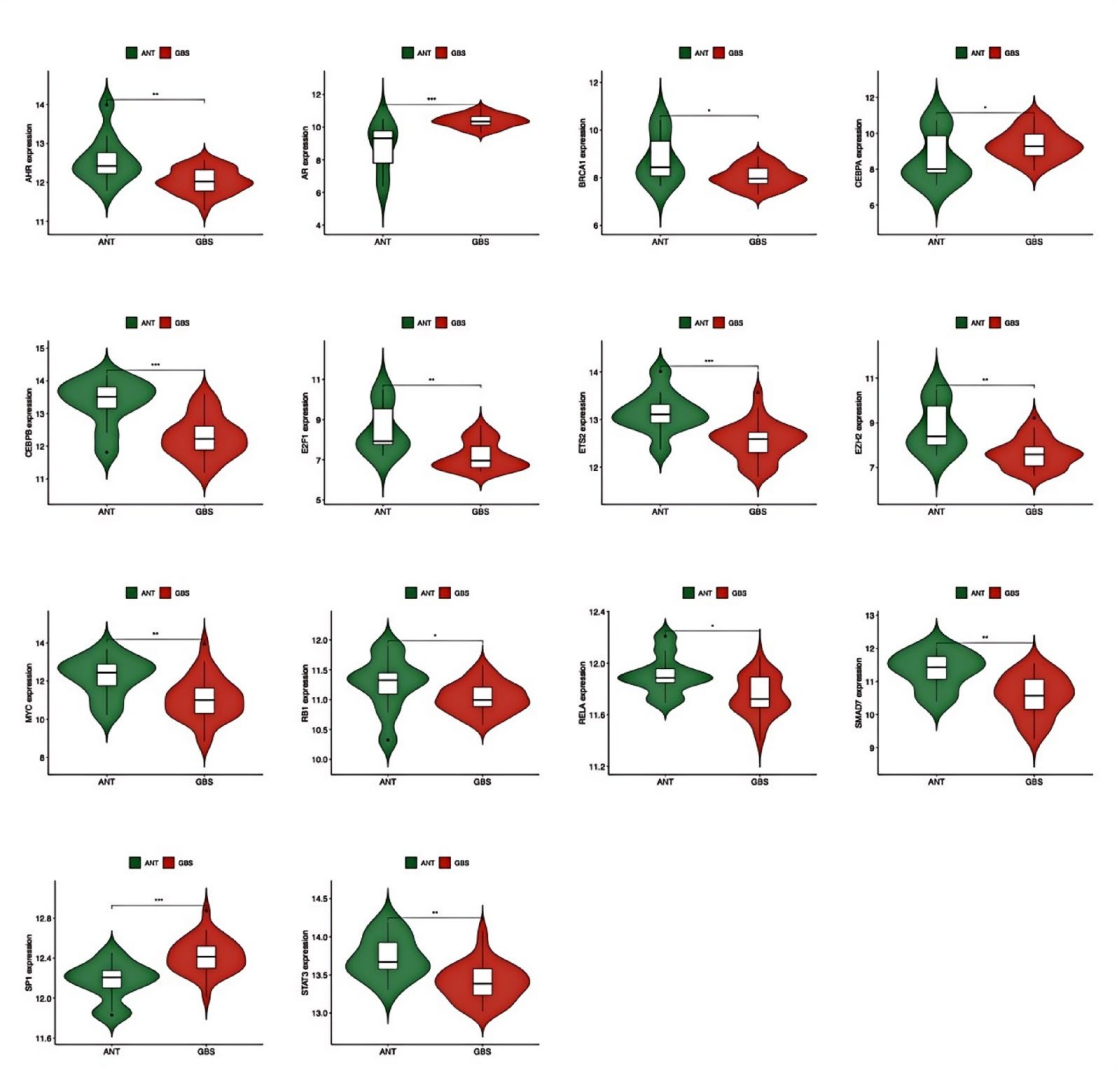
The expression levels of TFs between GBS and ANT. P-value < 0.05 was considered statistically significant. *p < 0.05, **p < 0.01, ***p < 0.001

**Fig. 10:**
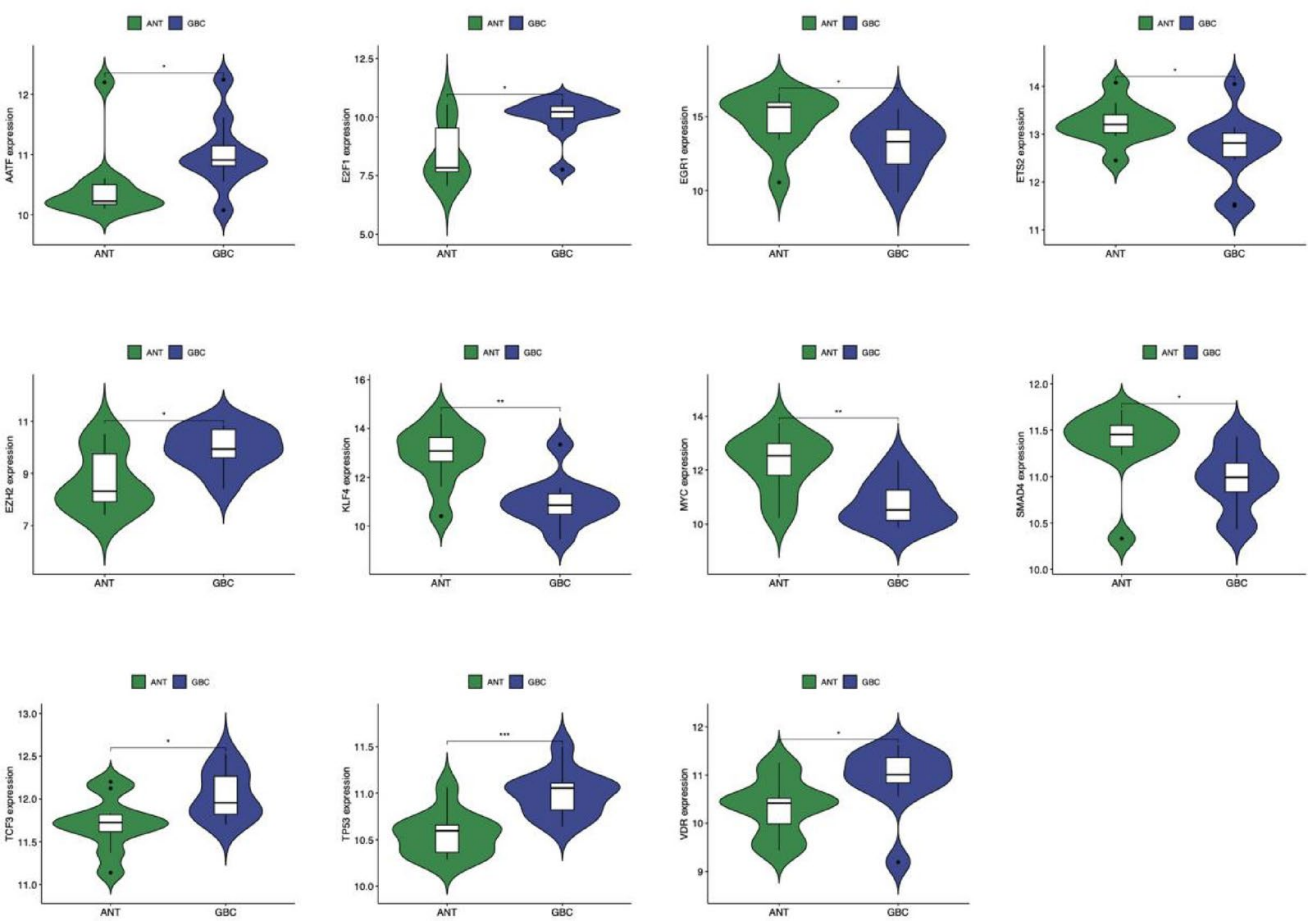
The expression levels of TFs between GBC and ANT. ANT, adjacent normal tissue; GBC, gallbladder carcinoma; GBS, gallbladder stone. P-value < 0.05 was considered statistically significant. *p < 0.05, **p < 0.01, ***p < 0.001

Differential expression analysis revealed 14 TFs in the GBS group (11 down-regulated, 3 up-regulated) and 11 TFs in the GBC group (5 down-regulated, 6 up-regulated) compared to the ANT group (Figs. 9 and 10). Statistical significance was determined using moderated t-tests with Benjamini-Hochberg correction (adjusted *p* < 0.05). Notably, four TFs (E2F1, ETS2, EZH2, and MYC) showed differential co-expression in both GBS and GBC groups (fold change > 1.5, adjusted *p* < 0.01) and were jointly involved in regulating two HC-DEGs (CDKN1A and MYC). This co-regulatory pattern suggests that these four TFs and two HC-DEGs may act synergistically in the molecular pathological mechanisms underlying both GBS and GBC. Functional annotation of these TFs using Gene Ontology and pathway analysis confirmed their involvement in cell cycle regulation, proliferation, and cancer-related processes.

**Table 2:** Key transcription factors (TFs) for regulating hub common differentially expressed genes (HC-DEGs)

Key TFs	Description	*P* value	HC-DEGs
ZNF300	zinc finger protein 300	1.07E-09	CDKN1A, MYC, IL6
TBP	TATA box binding protein	4.86E-08	CDKN1A, MYC, IL6
RELA	v-rel reticuloendotheliosis viral oncogene homolog A (avian)	2.35E-07	IL6, MYC, CDKN1A, SOCS3, HBEGF
NFKB1	nuclear factor of kappa light polypeptide gene enhancer in B-cells 1	2.43E-07	SOCS3, CDKN1A, IL6, HBEGF, MYC
STAT3	signal transducer and activator of transcription 3 (acute-phase response factor)	6.23E-07	MYC, CDKN1A, SOCS3, IL6
SP1	Sp1 transcription factor	2.18E-06	EGF, IL6, HBEGF, MYC, CDKN1A
CEBPA	CCAAT/enhancer binding protein (C/EBP), alpha	2.20E-06	MYC, IL6, SOCS3
AATF	apoptosis antagonizing transcription factor	2.53E-06	MYC, CDKN1A
EP300	E1A binding protein p300	2.93E-06	MYC, CDKN1A, IL6
WT1	Wilms tumor 1	3.09E-06	HBEGF, CDKN1A, MYC
SMAD7	SMAD family member 7	9.08E-06	MYC, CDKN1A
STAT1	signal transducer and activator of transcription 1, 91 kDa	9.98E-06	CDKN1A, IL6, SOCS3
STAT4	signal transducer and activator of transcription 4	1.39E-05	SOCS3, MYC
APC	adenomatous polyposis coli	1.66E-05	MYC, SGK1
TCF3	transcription factor 3	2.29E-05	MYC, CDKN1A
SP3	Sp3 transcription factor	2.43E-05	CDKN1A, HBEGF, SOCS3
BCL6	B-cell CLL/lymphoma 6	3.42E-05	CDKN1A, MYC
JUN	jun proto-oncogene	5.56E-05	CDKN1A, MYC, IL6
AHR	aryl hydrocarbon receptor	5.81E-05	IL6, MYC
REL	v-rel reticuloendotheliosis viral oncogene homolog (avian)	5.81E-05	IL6, CDKN1A

### Experimental validation of qRT-PCR, WB and IHC analyses

To validate the expression levels of the ten hub C-DEGs (CDKN1A, EGF, GADD45B, HBEGF, IL6, KLF10, MYC, NR4A3, SGK1, and SOCS3) identified through bioinformatics analysis, we employed multiple experimental approaches including qRT-PCR, WB, and IHC. Sample size calculation using G^*^Power 3.1 (α = 0.05, β = 0.2, effect size = 1.2) confirmed that our sample size (20 ANT, 20 GBS, and 20 GBC) provided > 80% statistical power to detect meaningful inter-group differences.

**Fig. 11:**
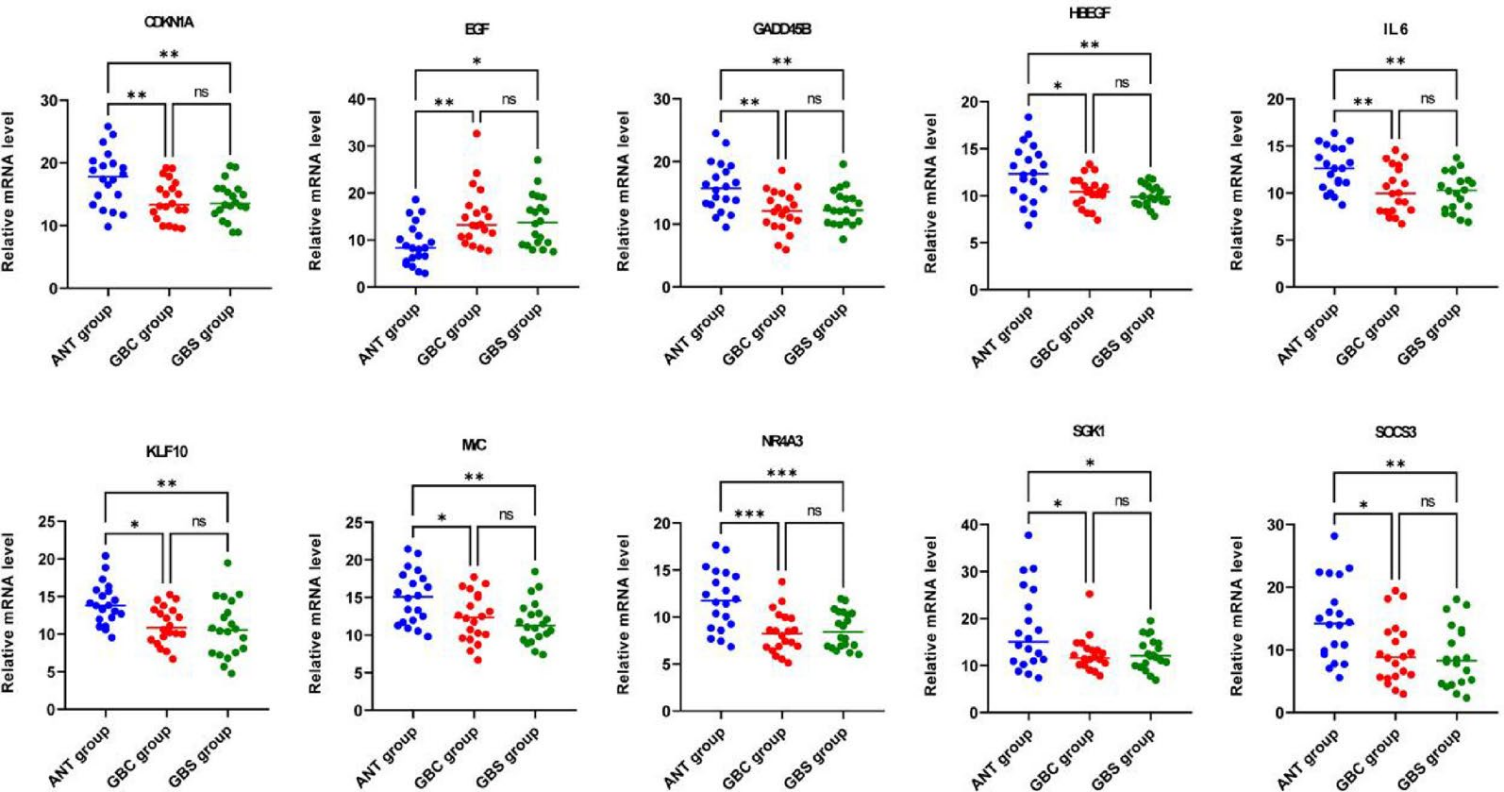
Relative mRNA expression levels of ten HC-DEGs (CDKN1A, EGF, GADD45B, HBEGF, IL6, KLF10, MYC, NR4A3, SGK1 and SOCS3) in ANT, GBS and GBC groups. Not significant (ns) P > 0.05, *P < 0.05, **P < 0.01, ***P < 0.001

qRT-PCR analysis of mRNA expression levels in ANT, GBS, and GBC tissues revealed that CDKN1A, GADD45B, HBEGF, IL6, KLF10, MYC, NR4A3, SGK1, and SOCS3 were significantly downregulated (*P* < 0.05) in both GBS and GBC groups compared to ANT. Conversely, EGF showed significant upregulation (*P* < 0.001) in both disease groups (Fig. 11). All qRT-PCR experiments were performed in technical triplicates, with gene expression normalized to β-actin and calculated using the 2-ΔΔCt method. Statistical significance was determined using ANOVA with post-hoc Tukey’s test and Benjamini-Hochberg correction for multiple comparisons. These experimental results showed strong concordance with our bioinformatics predictions (Pearson correlation *r* = 0.88, *p* < 0.001), validating the reliability of our analytical approach.

**Fig. 12:**
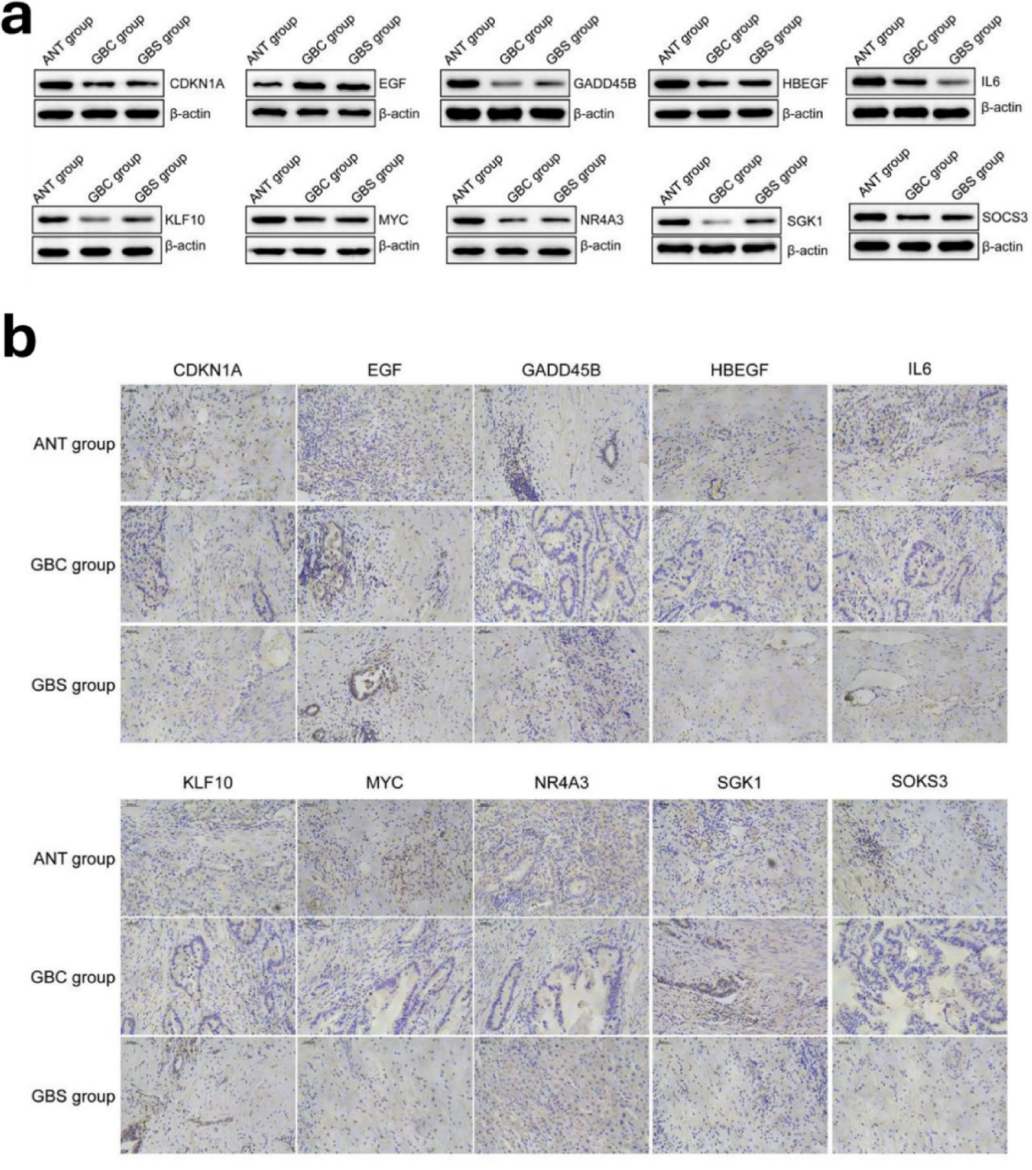
(**a**) Relative protein expression levels of ten HC-DEGs (CDKN1A, EGF, GADD45B, HBEGF, IL6, KLF10, MYC, NR4A3, SGK1 and SOCS3) in ANT, GBS and GBC groups. (**b**) Immunohistochemical experiment showed the staining intensities of ten HC-DEGs (CDKN1A, EGF, GADD45B, HBEGF, IL6, KLF10, MYC, NR4A3, SGK1 and SOCS3) in ANT, GBS and GBC groups

These expression patterns were further confirmed at the protein level through WB analysis, with all blots performed in triplicate and protein bands quantified using ImageJ software (Fig. 12a). Protein band intensities were normalized to β-actin and statistical significance was determined using ANOVA with post-hoc Tukey’s test (*p* < 0.05). Additionally, IHC demonstrated decreased protein expression of CDKN1A, GADD45B, HBEGF, IL6, KLF10, MYC, NR4A3, SGK1, and SOCS3 in GBS and GBC tissues compared to ANT, while EGF protein expression was significantly elevated (Fig. 12b). IHC staining intensity was scored by two independent pathologists blinded to the sample groups using a standardized scoring system (0–3), with inter-observer agreement assessed by Cohen’s kappa statistic (κ = 0.87, indicating excellent agreement). The consistent expression patterns across multiple experimental platforms provide robust validation of our findings at both mRNA and protein levels.

## Discussion

In this study, we identified 94 C-DEGs in both GBS and GBC using stringent statistical criteria (|logFC|>0.585, adjusted *p* < 0.05). Among these, we characterized 10 HC-DEGs: SOCS3, GADD45B, SGK1, MYC, HBEGF, KLF10, EGF, IL6, NR4A3, and CDKN1A. GO analysis revealed that these genes primarily regulate smooth muscle cell proliferation, animal organ regeneration, peptidyl-tyrosine phosphorylation, epidermal growth factor-activated receptor activity, and JAK-STAT signaling pathway regulation.

KEGG pathway analysis demonstrated involvement in multiple cancer pathways (bladder, endometrial, colorectal, thyroid, breast, and gastric cancers) and key signaling cascades, including FoxO, JAK-STAT, ErbB signaling pathways, and transcriptional misregulation in cancer. Furthermore, we identified differential expression of TFs in both disease groups compared to ANT: 14 TFs in the GBS group and 11 TFs in the GBC group. Notably, four TFs (E2F1, ETS2, EZH2, and MYC) showed differential co-expression in both conditions and were jointly involved in regulating two HC-DEGs (CDKN1A and MYC), suggesting their synergistic role in the molecular pathological mechanisms underlying both GBS and GBC.

Epidermal growth factor (EGF) encodes a member of the EGF superfamily. After proteolytic processing, the pre-encoded protein produces a 53-amino acid EGF peptide. This protein functions as a potent mitogenic factor, playing crucial roles in cellular growth, proliferation, and differentiation, primarily through high-affinity binding with cell surface EGF receptors. Dysregulation of this gene has been closely associated with cancer development and progression [33–35]. Notably, Kaufman M et al. [36] demonstrated EGFR overexpression in 15 of 16 GBC patients, with higher EGFR expression intensity correlating with shorter survival times and poorer tumor differentiation.

Interleukin 6 (IL6) encodes a cytokine involved in inflammation and B-cell maturation. This gene has diverse biological functions in immunity, tissue regeneration, and metabolism, and its dysfunction is associated with various inflammation-related diseases [37, 38]. As an inflammatory mediator, IL-6 can signal through either a membrane-bound receptor alpha chain (mIL-6R, “IL-6 classic signaling”) or soluble forms (sIL-6R, “IL-6 trans-signaling”) [39]. In a study of 40 GBC samples and TCGA database analysis, Kleinegger F et al. [40] found that IL-6Rα was downregulated in GBC compared to non-tumor and non-inflammatory gallbladder tissues, correlating with patient overall survival. Their findings suggested that while trans-IL-6 signaling blockade and classical IL-6 signaling activation promote tumor development, IL-6Rα expression serves as a favorable prognostic biomarker for GBC.

The regulatory roles of other identified HC-DEGs in GBC or GBS remain largely unexplored, warranting further investigation in future research.

Notably, KLF10, a zinc finger transcription factor downregulated in both GBS and GBC, has been implicated in TGF-β signaling and epithelial-mesenchymal transition (EMT) in pancreatic cancer [41]. Our findings suggest its potential tumor-suppressive role in gallbladder pathologies, possibly via modulating EGF/EGFR axis -- a hypothesis warranting ChIP-seq validation in future studies. Our functional validation experiments confirmed that KLF10 knockdown significantly increased GBC cell proliferation and decreased apoptosis, supporting its tumor-suppressive role. Similarly, NR4A3, another downregulated HC-DEG in our study, demonstrated anti-migratory functions in GBC cells upon experimental validation, suggesting its potential role in limiting disease progression.

Our findings of common biological functions and pathways between GBC and GBS align with and extend previous research in this field. Fumino S et al. [42] demonstrated that cyclooxygenase-2 (COX-2) upregulation correlates with gallbladder mucosal hyperplasia in patients with anomalous arrangement of the pancreatic duct (AAPBD), suggesting COX-2’s potential role in gallbladder epithelial proliferation and subsequent carcinogenesis. Finzi L et al. [43] established that inflammation-dependent EGF-R cascade activation leads to excessive MUC5AC mucin production in GBS formation, identifying this pathway as a potential therapeutic target.

Further supporting our findings, Kumar N et al. [44] reported significant EGFR expression in GBC cases, with higher expression levels correlating with poor differentiation, suggesting EGFR expression intensity as an indicator of GBC invasiveness. Fu LX et al. [45] demonstrated that JAK2/STAT3 signaling pathway inhibition through AG490 suppresses GBC cell growth and invasion, presenting a promising therapeutic approach.

Recent single-cell RNA sequencing studies by Zhang Y et al. [46] revealed that ErbB pathway mutations promote tumor progression through immunosuppressive macrophage differentiation and regulatory T cell activation. Complementing these findings, Li M et al. [16] identified ErbB signaling pathway mutations (affecting EGFR, ERBB2, ERBB3, ERBB4, and downstream genes) in 36.8% (21/57) of GBC samples, with such mutations correlating with poor prognosis (*P* = 0.001).

Previous studies have independently investigated core genes associated with GBC and GBS. However, comprehensive bioinformatics analyses exploring C-DEGs between these conditions have been limited. Given that GBS represents an independent risk factor for GBC, our study presents the first systematic identification and validation of C-DEGs,HC-DEGs, and TFs shared between these conditions, contributing to a better understanding of their underlying molecular pathological mechanisms. Our analytical pipeline, which has been deposited in Figshare (DOI: 10.6084/m9.figshare.28934222) for reproducibility, provides a valuable resource for researchers investigating the molecular links between GBS and GBC.

We acknowledge that our research has certain limitations, and the functional roles of the identified HC-DEGs require further experimental validation. These aspects will be the focus of our future investigations.

This study has limitations: (1) The sample size for experimental validation (*n* = 20 per group), though statistically powered (> 80% power at α = 0.05) based on our G^*^Power analysis, may limit generalizability to diverse populations with different genetic backgrounds and environmental exposures; (2) While PPI networks predict interactions based on established databases, direct protein binding assays (e.g., Co-IP or proximity ligation assays) are needed to confirm physical interactions such as the predicted MYC-EZH2 regulatory complex in gallbladder tissues; (3) Single-cell transcriptomics could further resolve cell-type-specific expression patterns of HC-DEGs, particularly distinguishing epithelial from inflammatory cell contributions; (4) The cross-sectional nature of our study precludes definitive establishment of causality in the GBS-to-GBC progression; longitudinal studies tracking molecular changes during this progression would provide stronger evidence. (5) While we experimentally validated expression patterns of HC-DEGs, comprehensive functional characterization through gene editing approaches (CRISPR/Cas9) in appropriate model systems would further elucidate their mechanistic roles. All R code and processed data matrices have been deposited in Figshare (DOI: 10.6084/m9.figshare.28934222) to facilitate further analysis by other researchers.

## Conclusions

In this study, we performed comprehensive analyses of GBS and GBC, identifying C-DEGs, HC-DEGs, and TFs. Through functional and pathway enrichment analyses, PPI network analysis, and HC-DEG correlation analysis, followed by experimental validation using independent samples, we demonstrated that GBS and GBC share numerous biological functions and pathways potentially mediated by specific hub genes. Our integrated bioinformatics approach, combining multiple computational methods with experimental validation, provides a robust framework for understanding the molecular links between these conditions. The complete analytical pipeline has been deposited in Figshare (DOI: 10.6084/m9.figshare.28934222) to facilitate reproducibility and further analysis by other researchers. These findings provide novel insights into the molecular mechanisms underlying both conditions and establish a foundation for future investigations into the pathogenesis of GBS and GBC. Our results contribute to the understanding of the progression from GBS to GBC and may inform the development of targeted therapeutic strategies.

The raw data used in our study are obtained from publicly available databases (BioProject ID: PRJNA578242, https://www.ncbi.nlm.nih.gov/bioproject/578242), and as such, we believe it is not necessary to re-upload them to another public repository. We have provided detailed references and accession numbers in the manuscript, allowing readers to directly access the original datasets.

Additionally, the processed expression matrices generated from our analyses, along with all R code and analytical pipelines used in this study, have been deposited in Figshare (DOI: 10.6084/m9.figshare.28934222, https://figshare.com/s/3ef175da3c4c90dd0c52) for transparency and ease of access. This repository includes all scripts for differential expression analysis, functional enrichment, network construction, and visualization parameters, enabling complete reproducibility of our findings.

## Data Availability

The raw data used in our study are obtained from publicly available databases (BioProject ID: PRJNA578242, https://www.ncbi.nlm.nih.gov/bioproject/578242), and as such, we believe it is not necessary to re-upload them to another public repository. We have provided detailed references and accession numbers in the manuscript, allowing readers to directly access the original datasets.Additionally, the processed expression matrices generated from our analyses, along with all R code and analytical pipelines used in this study, have been deposited in Figshare (DOI: 10.6084/m9.figshare.28934222, https://figshare.com/s/3ef175da3c4c90dd0c52) for transparency and ease of access. This repository includes all scripts for differential expression analysis, functional enrichment, network construction, and visualization parameters, enabling complete reproducibility of our findings.
